# Hidden features of NAD-RNA epitranscriptome in *Drosophila* life cycle

**DOI:** 10.1016/j.isci.2023.108618

**Published:** 2023-12-02

**Authors:** Shuwen Ge, Xueting Wang, Yingqin Wang, Minghui Dong, Dean Li, Kongyan Niu, Tongyao Wang, Rui Liu, Chao Zhao, Nan Liu, Ming Zhong

**Affiliations:** 1 Interdisciplinary Research Center on Biology and Chemistry, Shanghai Institute of Organic Chemistry, Chinese Academy of Sciences, 100 Hai Ke Road, Shanghai 201210, China; 2 University of Chinese Academy of Sciences, Beijing 100049, China; 3 Department of Critical Care Medicine, Zhongshan Hospital, Fudan University, Shanghai, China; 4 Shanghai Key Laboratory of Lung Inflammation and Injury, 180 Fenglin Road, Shanghai, China; 5 Shanghai Institute of Infectious Disease and Biosecurity, School of Public Health, Fudan University, Shanghai, China; 6 National Clinical Research Center for Aging and Medicine, Huashan Hospital, School of Basic Medical Sciences, Shanghai Medical College, Fudan University, 131 Dong An Road, Shanghai 200032, China; 7 Singlera Genomics, 500 Fu Rong Hua Road, Shanghai 201204, China; 8 Shanghai Key Laboratory of Aging Studies, 100 Hai Ke Road, Shanghai 201210, China

**Keywords:** Biological sciences, Genetics, Molecular genetics, Transcriptomics

## Abstract

Nicotinamide adenine dinucleotide (NAD), a nucleotide-containing metabolite, can be incorporated into the RNA 5′-terminus to result in NAD-capped RNA (NAD-RNA). Since NAD has been heightened as one of the most essential metabolites in cells, its linkage to RNA represents a critical but poorly studied modification at the epitranscriptomic level. Here, we design a highly sensitive method, DO-seq, to capture NAD-RNAs. Using *Drosophila*, we identify thousands of previously unexplored NAD-RNAs and their dynamics in the fly life cycle, from embryo to adult. We show the evidence that chromosomal clustering might be the structural basis by which co-expression can couple with NAD capping on physically and functionally linked genes. Furthermore, we note that NAD capping of cuticle genes inversely correlates with their gene expression. Combined, we propose NAD-RNA epitranscriptome as a hidden layer of regulation that underlies biological processes. DO-seq empowers the identification of NAD-capped RNAs, facilitating functional investigation into this modification.

## Highlights

• A much simplified and sensitive method, DO-seq, for NAD-RNA profiling
• Highly dynamic NAD-RNA epitranscriptome during *Drosophila* metamorphosis transition
• Co-expression coupled with NAD capping on functionally or physically linked genes
• NAD capping of cuticle genes is linked with adult transition of *Drosophila*

## Graphical abstract

**Figure:**
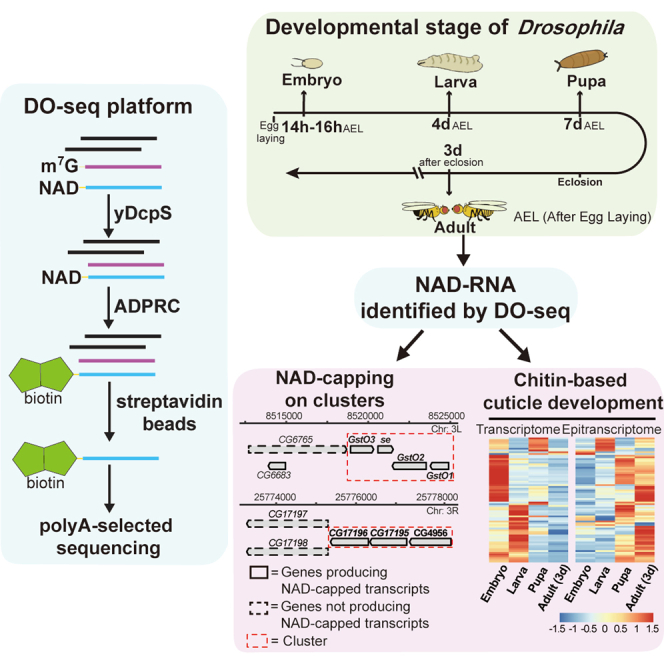

## Introduction

In eukaryotes, 5′,5′ -triphosphate-linked 7-methylguanosine (m^7^G) is the canonical 5′-end cap structure of RNA (m^7^Gppp-RNA or m^7^G-RNA), essential for the stability and biological function of mRNA species.^1^ NAD, the adenine nucleotide containing metabolite, emerged as a non-canonical initiating nucleotide (NCIN) incorporating at the 5′-terminus of RNA to result in NAD-capped RNA.^2^ Mass spectrometry analysis estimated that NAD modification constitutes approximately 0.6% and 1.3% of the mRNA transcripts from mouse liver and kidney, respectively.^3^ Since NAD capping is mediated by RNA polymerase II (RNA pol II), chromosomal clustered genes tend to be co-expressed in eukaryotes,^4^^,^^5^ prompting the question as to whether RNA pol II-mediated transcription and NAD capping can occur in physically-linked genes.

*Drosophila* life cycle begins in the form of embryo and subsequently undergoes a series of physical transformations until reaching adulthood. Such dramatic changes are called metamorphosis. Mounting evidence associates fly developmental transitions with transcriptional and metabolic changes,^6^^,^^7^ yet how these processes are integrated into the regulation of fly life cycle is only beginning to be revealed. NAD-functionalized RNAs, poised to integrate metabolome and transcriptome, might represent novel epitranscriptomic mechanisms. However, the unknown identity of NAD-RNAs has hindered functional studies.

The previously reported transcriptome-wide NAD-RNA profiling method depends on multiple chemo-enzymatic reactions and high RNA input.^8^^,^^9^^,^^10^ NAD captureSeq was initially reported, combining an enzymatic reaction by adenosine diphosphate ribosyl-cyclase (ADPRC) and copper-catalyzed click chemistry to biotinylate NAD-RNAs for affinity binding.^8^ However, the presence of copper ions led to RNA degradation.^8^ SPAAC-NAD-seq employed the copper-free strain-promoted azide alkyne cycloaddition (SPAAC) to preserve RNA integrity.^9^ Since ADPRC has been noted to catalyze the reaction with m^7^G-RNAs, SPAAC-NAD-seq requires antibody based pre-treatment to avoid false-positive hits derived from m^7^G-capped transcripts, thus demanding additional procedures and high RNA input.^9^ Recently we reported ONE-seq method, utilizing a one-step enzymatic reaction by ADPRC and HEEB (N-[2-(2-hydroxyethoxy) ethyl]-biotinamide) as a reactant to biotinylate NAD-RNAs for streptavidin affinity binding.^11^ ONE-seq employs a NudC-based post-treatment to elute biotin-conjugated RNAs specifically derived from NAD, but not m^7^G-capped transcripts from streptavidin beads.^11^ In the present study, we design the DO-seq method by introducing yDcpS-based pre-treatment to remove m^7^G cap prior to the ADPRC reaction, thereby allowing increased amount of HEEB reactant to further improve the capture sensitivity. Using *Drosophila*, we proceed to explore the utility of DO-seq and reveal novel insights of NAD capping events in the fly life cycle.

## Results

### The workflow of DO-seq

**Figure 1:**
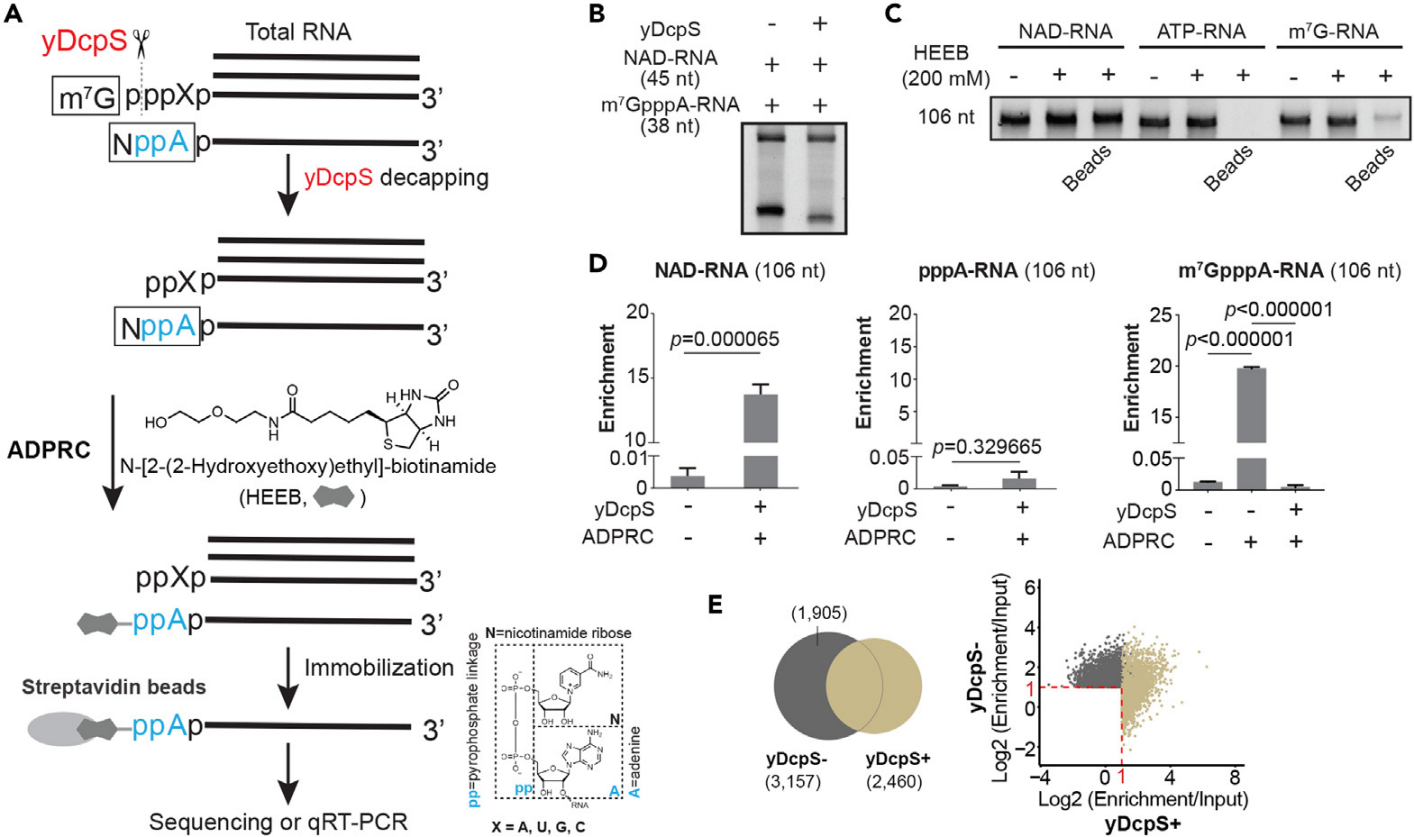
The workflow and specificity of DO-seq (A) The workflow of DO-seq: First, yDcpS (in red) de-caps the m^7^G moiety from m^7^G-RNAs in total RNAs. Second, in the presence of ADPRC, NAD-RNAs can be biotinylated by HEEB, which subsequently are enriched by streptavidin beads. Enriched RNAs can be used for epitranscriptome-wide profiling as well as qRT-PCR analysis. (B) yDcpS was able to de-cap m^7^GpppA-RNA (38 nt) but not NAD-RNA (45 nt), as evidenced by a lower-sized band corresponding to the de-capped product in the TBE-Urea gel. (C) HEEB (200 mM) reacted with NAD-RNA (106 nt) and m^7^GpppA-RNA (106 nt), but not pppA-RNA (106 nt), resulting in a band retained by the streptavidin beads. (D) qRT-PCR analysis showed that NAD-RNA (106 nt), but not pppA-RNA (106 nt), could be enriched by DO-seq. qRT-PCR analysis showed that m^7^GpppA-RNA (106 nt) could be detected in the presence of ADPRC; pre-treatment of yDcpS eliminates potential enrichment of m^7^GpppA-RNA (106 nt). The difference between Ct of target RNA in enrichment and input was calculated, resulting in index conversion of ΔCt. Data were shown in mean ± s.e.m. (E) Epitranscriptome assessment of yDcpS to minimize the noise of m^7^G-RNAs. Two-fold enrichment of read counts was used as the cutoff. Standard DO-seq identified 2,460 NAD-RNAs, while 1,905 false-positive NAD-RNAs were found without the use of yDcpS, presumably derived from m^7^G-capped RNAs. Total RNAs were from liver tissues of 18-month mice.

To further enhance the capture efficiency, we introduced the yDcpS enzyme, known for its ability to de-cap m^7^G, but not NAD from RNA transcripts. We thereby named our method DO-seq, through y**D**cpS pre-treatment followed by **O**ne-step chemo-enzymatic reaction to biotinylate NAD-capped RNA for high-throughput **seq**uencing (Figure 1A). By pre-clearance of m^7^G cap from total RNA, DO-seq allows increased HEEB reactant to promote biotinylation.

### Specificity of DO-seq

We tested the specificity of yDcpS. First, we showed the evidence that yDcpS was able to de-cap m^7^GpppA-RNA (38 nt) but not NAD-RNA (45 nt) synthetic RNA oligonucleotides as demonstrated by a lower-sized band in the TBE-Urea gel corresponding to the de-capped product (Figure 1B). Second, we used 200 mM HEEB, an amount two times higher than that used in our previous study.^11^ To test specificity, we generated three groups of 106 nt synthetic RNA spike-ins (i.e., pppA-RNA without a cap, NAD-capped RNA, and m^7^G-capped RNA (m^7^GpppA-RNA)). HEEB (200 mM) reacted with NAD-RNA (106 nt) and m^7^GpppA-RNA (106 nt), but not pppA-RNA (106 nt), resulting in a band retained by the streptavidin beads (Figure 1C). Since biotinylated m^7^GpppA-RNA, even at a low level, would cause false-positive signals, this result highlights the necessity of pre-clearance of m^7^G capped RNA transcripts. We further subjected total RNA extract mixed with spike-in RNAs to the DO-seq experiment, followed by qRT-PCR. NAD-RNA (106 nt), in an ADPRC-dependent manner, was robustly and significantly enriched, while pppA-RNA (106 nt) could not be detected (Figure 1D). In the presence of ADPRC, m^7^GpppA-RNA (106 nt) could be detected; treatment of yDcpS, however, eliminated potential enrichment of m^7^GpppA-RNA (106 nt) (Figure 1D). Third, we determined the noise-canceling effect of yDcpS by comparative analysis of RNA-seq datasets. Total RNAs, either mock-treated or yDcpS-treated, were subjected to HEEB reaction, followed by enrichment via streptavidin beads (Figure S1A). polyA-selected RNA sequencing was performed. Principal component analysis revealed that the biological replicates of each library type clustered together, indicating the high quality and reproducibility of the sequencing data (Figure S1B). The distance between the mock-treated and yDcpS-treated samples implied that the identified NAD-RNAs differed upon yDcpS treatment (Figure S1B). To pinpoint NAD-capped RNA, we set 2-fold enrichment of read counts as the cutoff (Figure S1A). In total, from mouse liver tissues, 3,157 NAD-RNAs were identified in the absence of yDcpS; however, the usage of yDcpS expelled 1,905 genes from the profile (Figure 1E; Table S1). As a result, yDcpS treatment may contribute by roughly reducing 60% of the false positive hits presumably derived from m^7^G-capped RNAs. To assess the specificity of the capture procedure, we subjected total RNA from mouse livers mixed with spike-in RNAs to yDcpS pre-treatment with or without ADPRC treatment, followed by streptavidin beads enrichment and qRT-PCR (Figure S1C). Notably, in the presence of ADPRC, NAD-RNA spike-in (500 nt) and endogenous genes identified as NAD-RNAs, such as Akr1c20, Apom, C1d, and Dbi, but not ppp-RNA (500 nt) and m^7^G-RNA (500 nt) spike-ins, showed significant enrichment, whereas none of these transcripts could be enriched in the absence of ADPRC (Figure S1D).

### Sensitivity of DO-seq

**Figure 2:**
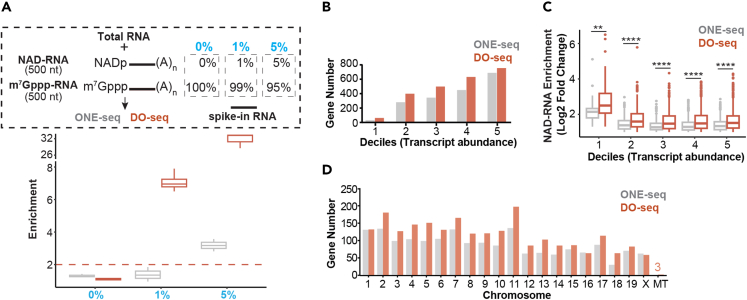
Sensitivity of DO-seq (A) RNA-seq experiment of spike-in RNAs determined the sensitivity of DO-seq. Top panel: schematic workflow of total RNAs and polyadenylated spike-in RNAs that have different ratios of NAD-RNA (500 nt). Two spike-in RNAs with identical sequence (500 nt) but have either NAD or m^7^G-cap, followed by polyA tails are used; bottom panel: fold change of normalized read counts from spike-in RNA between enrichment and input samples in different ratios of NAD-RNA. Total RNAs were from liver tissues of 2-month male mice. The nominal ratios of NAD-RNAs were highlighted in blue. Boxplot showed the first quartile, median and third quartile, and whiskers represented 1.5x interquartile ranges. (B) More NAD capping events could be identified by DO-seq than ONE-seq. (C) More NAD capping events could be identified by DO-seq than ONE-seq, even for lowly expressed transcripts. (Two-tailed Student’s *t* test: ∗∗∗∗p < 0.0001; ∗∗p < 0.01). (D) DO-seq was able to capture three NAD-RNAs encoded by the mitochondrial genome.

We examined the sensitivity of DO-seq by comparing it with ONE-seq, our recently reported method. First, we used RNA spike-ins to assess the capture sensitivity of the two methods. We synthesized two long RNA spike-ins with identical sequence (500 nt) but had either NAD or m^7^G-cap, followed by polyA tails. Presumably, endogenous transcripts may contain both NAD and m^7^G-capped forms, though the percentage may differ for particular genes. The rationale of this design is to mimic endogenous genes that have either low (0% or 1%) or relatively high (5%) NAD modification. Total RNAs were mixed with spike-ins that had different ratios of NAD-RNA and m^7^G-RNA. The sample that contained 100% m^7^G-RNA spike-in represented a gene with no NAD capping, which allowed the assessment of the specificity of the capture method. Other samples that contained 1% and 5% of NAD-relative to m^7^G-RNA spike-ins were used to determine the capture sensitivity. We subjected 100 μg total RNA from mouse livers mixed with 1 ng spike-in RNA to either DO-seq or ONE-seq methods, followed by polyA-selected RNA sequencing. In the sample mixed with 100% m^7^G-RNA spike-in, we found no enrichment for both methods (Figure 2A), highlighting the specificity. In the sample that contained 5% of NAD-capped forms, DO-seq showed higher enrichment level (31-fold) than ONE-seq (3.4-fold) (Figure 2A). When NAD-capped forms accounted for 1% of the transcript, the enrichment was low (1.5-fold) and variable for ONE-seq (Figure 2A); in sharp contrast, DO-seq obtained a remarkable 7-fold enrichment. Second, we assessed NAD-RNA epitranscriptome profiles obtained by DO-seq and ONE-seq. In general, more NAD capping events could be identified by DO-seq than ONE-seq (Figure 2B), particularly for lowly expressed transcripts (Figure 2C). Moreover, DO-seq, but not ONE-seq, was able to capture three NAD-RNAs encoded by the mitochondrial genome (Figure 2D). Together, these data suggest that DO-seq outperforms ONE-seq in terms of capture sensitivity while ensuring specificity.

### Epitranscriptomic profiling of *Drosophila* life cycle, from embryo to adult

**Figure 3:**
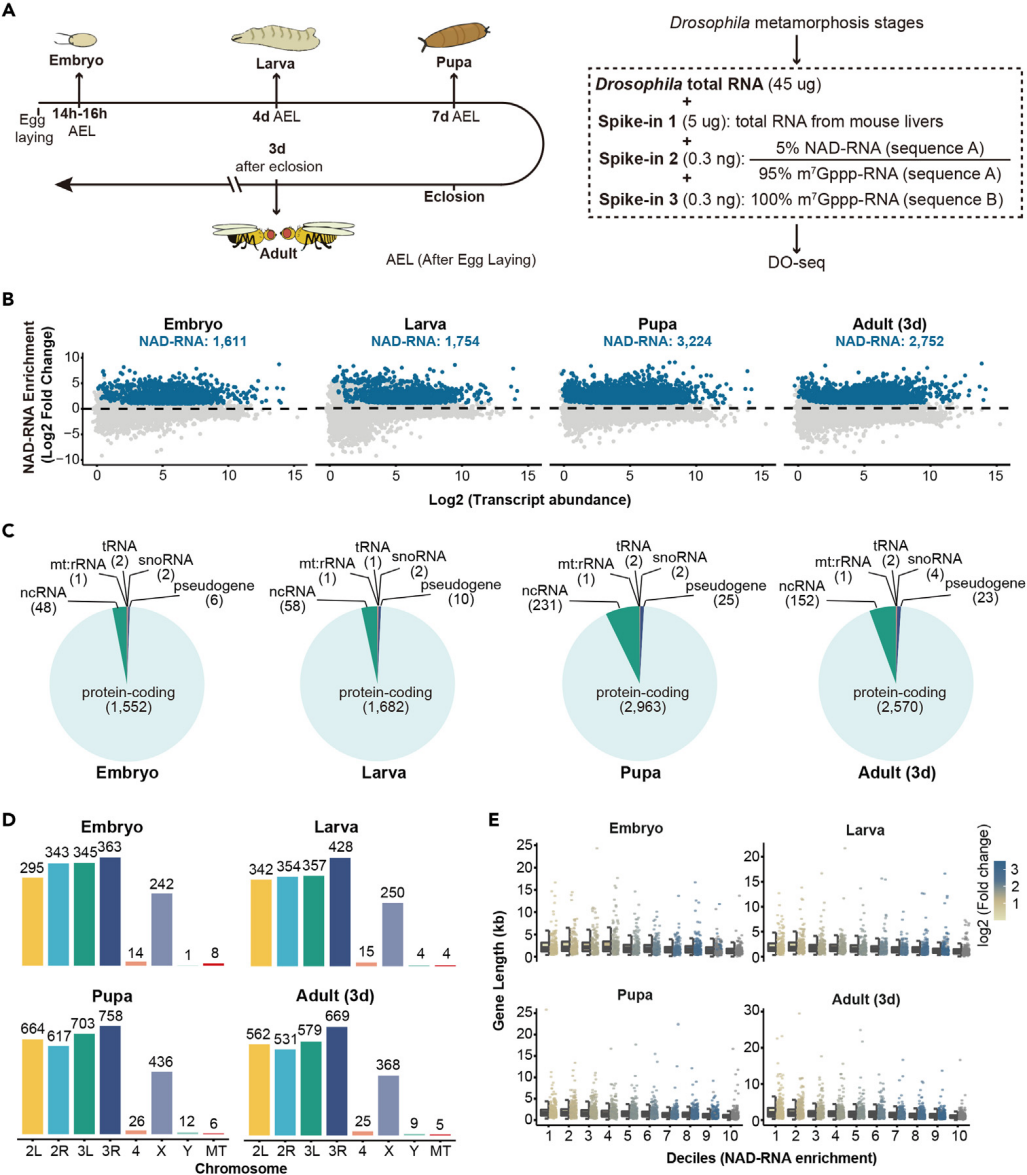
Epitranscriptome-wide profiling of NAD-RNAs in *Drosophila* life cycle, from embryo to adult (A) Experiment outline. To fully profile NAD-RNAs from *Drosophila* life cycle, samples from four stages (embryo (14h–16h AEL), larva (4days AEL), pupa (7days AEL) and adult fly (3days)) were collected. Total RNAs from those samples were mixed with spike-in RNA from mouse livers, and two synthetic spike-in, of which one with 5% NAD-capped forms and another with 100% m^7^G-capped forms. The mixtures were then subjected to DO-seq. (B) Scatterplots showing NAD-RNAs (blue dots) identified by DO-seq at four stages. 2-fold enrichment of read counts was used as the cutoff. 1,611, 1,754, 3,224 and 2,752 NAD-RNAs were identified from *Drosophila* embryo, larva, pupa, and adult (3days), respectively. (C) NAD capping mostly occurred on protein-coding genes but also extended to pseudogenes and ncRNA. (D) Chromosomal distribution showed that NAD-RNAs were derived from genes localized on all three autosomes, and both X and Y chromosomes as well as the mitochondrial genome. (E) From 10 deciles based on enrichment, shorter genes tended to have increased modification of NAD. Boxplot showed the first quartile, median and third quartile, and whiskers represented 1.5x interquartile ranges.

To study how metabolite-modified RNA transcripts modulate complex biological processes, we profiled NAD-RNAs from *Drosophila* life cycle, including highly dynamic metamorphosis transitions from embryo, larva, pupa to mature adult at the 3days of age (Figure 3A). We recently developed a spike-in-based normalization and data-driven evaluation framework, enONE, for the omic-level analysis of NAD-capped RNAs.^12^ Toward quantitative assessment, we deliberately included three types of spike-in RNAs: (1) total RNAs from mouse livers, a vertebrate model organism with well-annotated genome sequence, were used to normalize technical variations among independent biological repeats; (2) synthetic RNAs, consisting of 5% NAD-relative to m^7^G-capped forms, were used to determine the capture sensitivity; (3) synthetic RNAs, with 100% m^7^G-capped forms, were used to determine the capture specificity (Figure 3A). Spike-ins 2 and 3 were synthesized with templates from different sequences. Combined, we subjected 45 μg total RNA from *Drosophila* mixed with 5 μg total RNA from mouse liver, 0.3 ng spike-in 2 RNA, and 0.3 ng spike-in 3 RNA to the DO-seq experiment, followed by enONE computational analysis.

After quality control, we obtained an average ∼5.3 million high-quality and uniquely mapped sequencing read pairs from each library (Figure S2A). Assessment of datasets corroborated that sequencing saturation has been reached (Figure S2B). Spike-in 2, which contained 5% NAD-capped forms, were significantly enriched, whereas no enrichment was found for spike-in 3 made up with 100% m^7^G-RNA (Figure S2C). The above evidence highlighted the sensitivity and specificity of DO-seq, as reflected by the enrichment of NAD, but not m^7^G, capped transcripts. Upon enONE normalization, principal component analysis (PCA) illustrated that the biological replicates of each sample type clustered together, reflecting high reproducibility of the experiments (Figure S2D). Given the specificity of our capture strategy, we proceeded to set 2-fold enrichment of read counts as the cutoff, which led us to identify 1,611, 1,754, 3,224, and 2,752 NAD-capped RNA transcripts from *Drosophila* embryo, larva, pupa, and adult, respectively (Figure 3B; Table S2). Notably, the number of NAD-RNAs progressively increased during development, peaking at the pupa stage and then slightly dropped in adult flies. Moreover, we also provided lists of NAD-RNAs with fold enrichment at and higher than 4, 6, and 8, respectively (Table S2).

We validated newly identified NAD-RNAs using a slightly modified CapZyme-Seq method.^13^ Briefly, total RNAs from adult flies were pre-treated with calf-intestinal alkaline phosphatase (CIAP) to eliminate phosphate groups from the 5′ end of non-capped RNAs, followed by the treatment of NudC pyrophosphatase that efficiently hydrolyzes NAD-capped RNA, generating a ligatible 5′ monophosphate on the RNAs. These RNAs were subject to adapter ligation, adapter-based cDNA synthesis, and PCR amplification (Figure S3A). Control studies demonstrated that NAD-RNA (500 nt) spike-ins, but not ppp-RNA (500 nt) and m^7^Gppp-RNA (500 nt), were selectively and significantly enriched (Figure S3B), suggesting the feasibility of this validation method. We subsequently examined the NAD capping for four *Drosophila* endogenous transcripts, S1P, mt:srRNA, TwdlL, and TwdlG, as identified by DO-seq (Figure S3B). By contrast, two genes that were not identified by DO-seq, RpL9 and CG11454, showed no enrichment (Figure S3B). Combined, this validation corroborated newly identified NAD-RNAs, supporting the notion that DO-seq allows robust and efficient identification of NAD-RNAs.

### Characterization of NAD-RNAs from *Drosophila*

**Figure 4:**
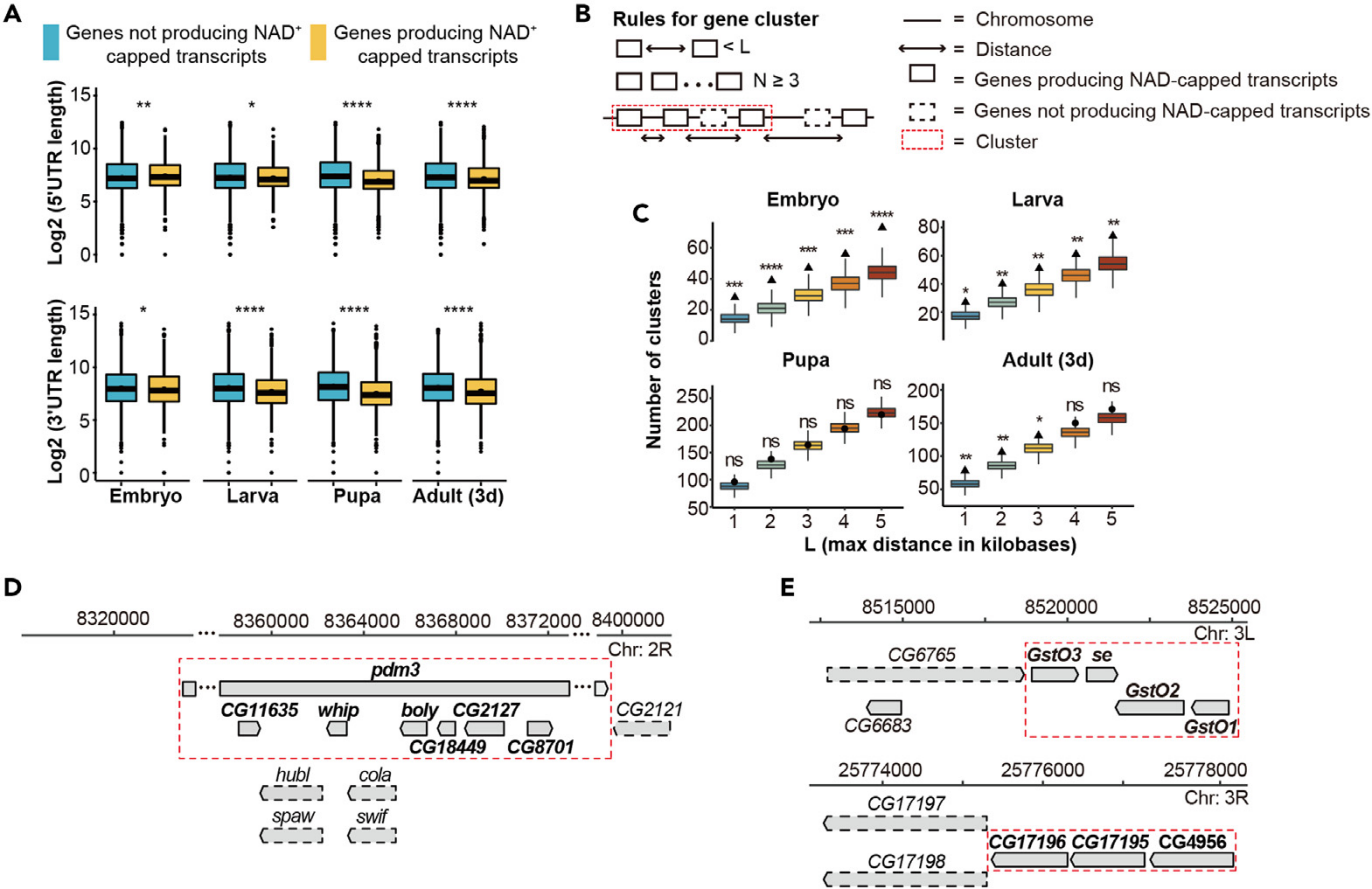
Structural features of genes producing NAD-RNAs (A) At the embryonic stage, NAD-RNAs tended to have longer 5′ UTR and shorter 3' UTR compared to those only capped by m^7^G. From larval to early adult, transcripts producing NAD cap appeared to have shorter 5′ UTR and 3' UTR than those not producing NAD cap. Boxplot showed the first quartile, median and third quartile, and whiskers represented 1.5x interquartile ranges. (Two-tailed Student’s *t* test: ∗∗∗∗p < 0.0001; ∗∗p < 0.01; ∗p < 0.05). (B) Schematic view of identification of physical clusters. A physical cluster is defined as a group of ***N*** (***N*** ≥ 3) genes producing NAD-capped transcripts where the distance between two adjacent genes was not over ***L*** kilobases. The distance was calculated as the difference between the stop position of the most 5′ gene and the start position of the most 3′ gene. (C) Significantly more gene clusters were found than expected by random during *Drosophila* development, except for the pupa stage (∗∗∗∗p < 0.0001; ∗∗∗p < 0.001; ∗∗p < 0.01; ∗p < 0.05; n.s., not significant). Different settings for ***L*** [1, 2, 3, 4, 5 kb] were tested. The null distributions, obtained from stimulations, are shown as a boxplot. The actual number of clusters found is marked with a filled dot (not significant) or triangle (significant), depending on the p-value assigned by the corresponding null distribution. A close-up of (D) the largest cluster and (E) two clusters of functionally-linked genes. The chromosomal region was indicated by position coordinates. Genes were indicated as a directional rectangle. Surrounding solid line: Genes producing NAD-capped transcripts; Surrounding dashed line: genes not producing NAD-capped transcripts. Clusters were indicated as rectangles surrounding red dashed lines.

We characterized newly identified NAD-RNAs from *Drosophila*. In flies, NAD-capping mostly occurred on protein-encoding genes, but also extended to pseudogenes and non-coding RNAs (ncRNA), including snoRNAs, antisense RNAs, and lncRNAs (Figure 3C). Pupa appeared to contain the most abundant NAD capping on ncRNAs (Figure 3C), compared to flies at other stages. NAD-RNAs were shown to be derived from genes localized on all three autosomes, even *Drosophila* chromosome 4, and both X and Y chromosomes as well as the mitochondrial genome (Figure 3D). By dividing NAD-RNAs into 10 deciles based on enrichment, we observed that shorter genes tended to have increased modification of NAD (Figure 3E), consistent with previous findings in *Arabidopsis* and mouse.^11^^,^^14^ Furthermore, we observed a feature in the length of the untranslated regions (UTRs) of NAD-RNAs. At the embryonic stage, NAD-RNAs tended to have longer 5′ UTR and shorter 3' UTR compared to those only capped by m^7^G (Figure 4A). From larval to early adult, however, transcripts producing NAD cap appeared to have shorter 5′ UTR and 3' UTR than those not producing NAD cap (Figure 4A).

### Coordinated NAD capping of physically linked genes

We studied whether NAD modification occurred in physically-linked genes. To address this, we analyzed gene clusters that co-expressed at the level of transcriptome and were also enriched by DO-seq reflective of NAD capping. A physical cluster was formed and iteratively expanded with the closest genes if the genomic distance was not over maximum distance ***L*** (Figure 4B). Different settings for ***L*** [1, 2, 3, 4, 5 kb] were tested. Remarkably, significantly more gene clusters were found than expected by random, except for the pupa stage (Figure 4C). At the setting of ***L*** = 3 kb, we obtained significant clustering with the most number and size, i.e., 47, 51, 164, and 131 clusters that contained NAD-capped genes at the embryo, larva, pupa, and adult flies, respectively (Table S3). The largest cluster was at the location of *pmd3* with six genes being co-expressed and NAD-capped (Figure 4D). Moreover, functionally similar genes were found to be not just physically clustered but also capped by NAD, e.g., Glutathione S-transferase family (*GstO1*, *GstO2*, *GstO3*, *se*) and palmitoyltransferases family (*CG17195*, *CG17196*, *CG4956*) (Figure 4E). Combined, our study indicates that chromosomal clustering might provide a structural basis by which co-expression can couple with NAD capping on functionally or physically linked genes.

### NAD capping of cuticle genes underlying adult transition

**Figure 5:**
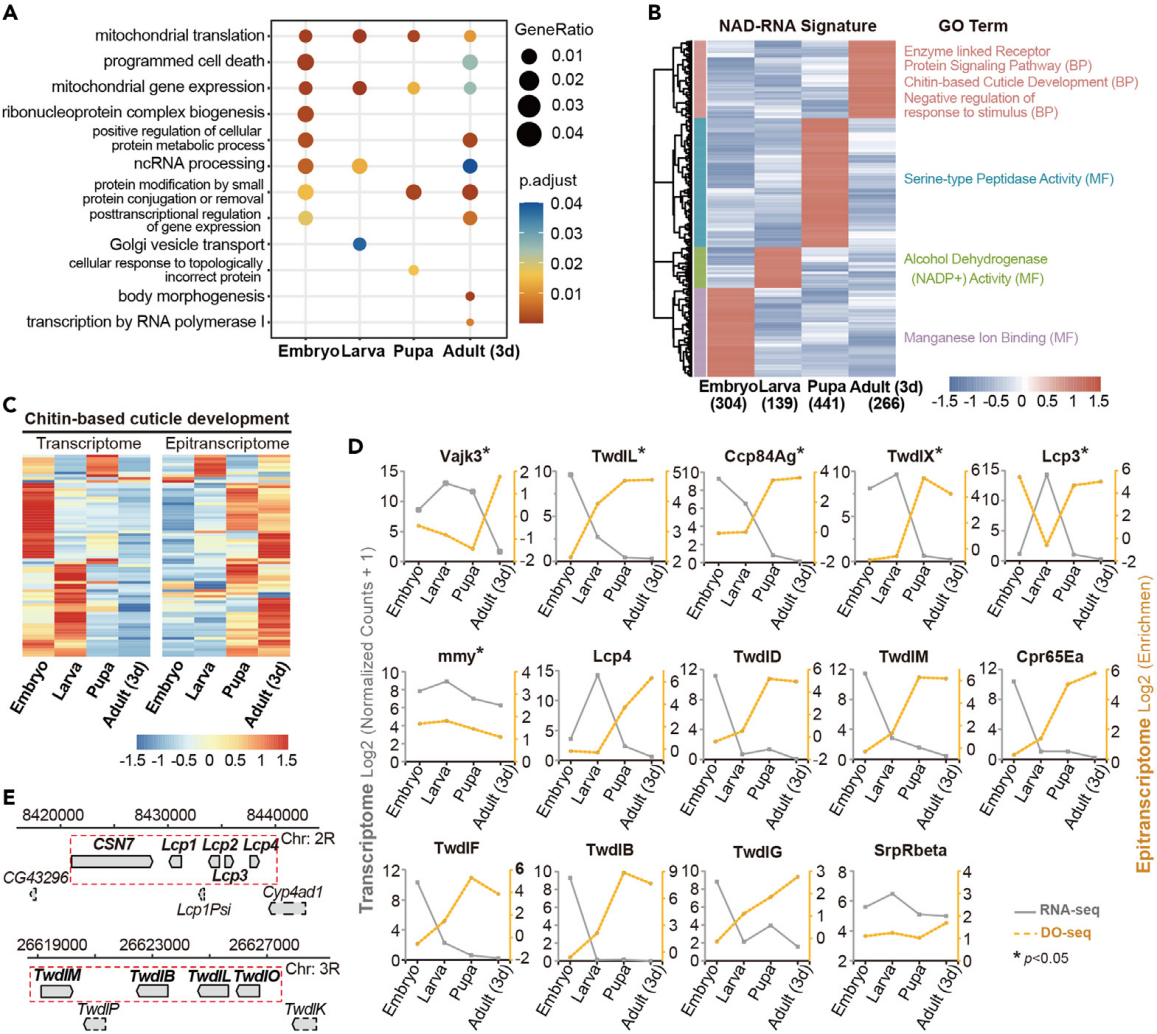
Functional relevance of highly dynamic NAD-modified epitranscriptomes in *Drosophila* (A) Pathway analysis revealed that mitochondrial translation and mitochondrial gene expression were cellular events enriched for NAD capping throughout *Drosophila* life cycle. Stage-specific biological processes were also illustrated. (B) Heatmap showing epitranscriptome signatures identified by using *Z* score >1.3. 304, 139, 441, and 266 NAD-RNAs were identified as epitranscriptome signatures with their distinguished expression for each stage. Pathway analysis noted that chitin-based cuticle development was highlighted as an NAD-RNA signature in the adult. (C) Heatmap showing a reciprocal correlation between transcriptome and epitranscriptome in 72 genes of chitin-based cuticle development. (D) Double yaxis graph illustrating the opposite trend in gene expression and NAD modification of a single gene during *Drosophila* development. Significances (Pearson correlation analysis, ∗p < 0.05) were reached for six genes, with five genes (*Vajk3*, *TwdlL*, *Ccp84Ag*, *TwdlX*, *Lcp3*) being negative correlations and only one (*mmy*) being positive relation. The left axis showed the normalized read counts in RNA-seq and the right axis marked the fold change of normalized read counts between enrichment and input. Data were shown in log2-transformed format. (E) A close-up of two clusters of genes related to chitin-based cuticle development. The chromosomal region was indicated by position coordinates. Genes were indicated as a directional rectangle. Surrounding solid line: Genes producing NAD-capped transcripts; Surrounding dashed line: genes not producing NAD-capped transcripts. Clusters were indicated as rectangles surrounding red dashed lines.

We aimed to reveal new features defined by NAD-RNA epitranscriptome. Pathway analysis revealed that mitochondrial translation and mitochondrial gene expression were cellular events enriched for NAD capping throughout *Drosophila* life cycle, from embryo to adulthood. Stage-specific biological processes such as ribonucleoprotein complex biogenesis, Golgi vesicle transport, body morphogenesis, and etc., were highlighted (Figure 5A). Moreover, 43 genes encoding transcription factor and 36 for co-factors were found to be capped by NAD (Figure S4; Table S4). These data indicate highly dynamic NAD-modified epitranscriptomes and their functional relevance during *Drosophila* development.

Using *Z* score >1.3, we further identified 304, 139, 441, and 266 NAD-RNAs as epitranscriptome signatures with their distinguished expression for each stage (Figure 5B). Similarly, transcriptome signatures were obtained from gene expression profiles (Figure S5A). Interestingly, pathway analysis noted that chitin-based cuticle development was highlighted as NAD-RNA signature in the adult (Figure 5B), whereas this pathway was enriched as transcriptome signature from embryo to pupa, except for adulthood. (Figure S5B). *Drosophila* genome is known to encode 201 genes of chitin-based cuticle development,^15^ with 72 of them being found to be capped by NAD. Stage-specific inspection revealed that their expression at the RNA level was relatively high at the embryo and larva stages when NAD capping was low, but later became downregulated at both pupa and adult when NAD capping was increased, thus displaying a reciprocal manner (Figure 5C). Significances (Pearson correlation analysis, p < 0.05) were reached for six genes, with five genes (*Vajk3*, *TwdlL*, *Ccp84Ag*, *TwdlX*, *Lcp3*) being negative correlation and only one (*mmy*) being positive relation (Figure 5D). Other genes in the same pathway, though not statistical significance, demonstrated opposite trends in gene expression and NAD modification (Figure 5D). Moreover, we found that coordinated NAD modification of physically-linked genes also extended into two gene clusters of chitin-based cuticle development, one from the CPR family (*Lcp1*, *Lcp2*, *Lcp3*, *Lcp4*), and another from the Tweedle family (*TwdlM*, *TwdlB*, *TwdlL*, *TwdlO*) (Figure 5E), providing evidence of evolutionary convergence that integrates chromosomal clustering of functionally linked genes and NAD capping.

## Discussion

NAD is the hub metabolite and redox regent for cells, essential for a wide range of biological processes.^16^^,^^17^ The covalent bonding of NAD to RNA essentially connects metabolic regulation with gene expression, representing a critical but poorly-studied modification at the epitranscriptomic level. However, investigating biological insight of NAD-RNAs has been hindered by the profiling methods available. We recently devised the ONE-seq method, through a one-step chemo-enzymatic reaction by ADPRC and HEEB as a reactant to directly capture NAD-RNAs from total RNAs.^11^ To avoid m^7^G-contaminating signals, ONE-seq used 100 mM HEEB for the reaction followed by a NudC-based post-treatment to specifically harvest NAD-RNAs.^11^ In the present study, we design the DO-seq method by yDcpS-based pre-treatment to remove m^7^G cap prior to the ADPRC reaction, thereby allowing increased amount of HEEB reactant (200 mM) to further improve the capture sensitivity while ensuring specificity. Compared to ONE-seq, DO-seq is able to lower the detection limit to 1% of NAD-capped form for a particular transcript. Consequently, DO-seq significantly expands the horizon of NAD-RNA epitranscriptome, especially for lowly expressed transcripts as well as mitochondria-encoded genes. Since NAD modification was originally reported in the RNA species from prokaryotic organisms where m^7^G cap was absent^18^^,^^19^ and mitochondria are known to evolve from endosymbiotic bacteria, NAD capping that occurs in both mitochondria and host transcriptomes highlights the evolutionary consensus and further supports the notion that NAD-RNA defines a fundamental regulatory mechanism at the epitranscriptomic level.

Empowered by DO-seq, we are able to identify thousands of previously unexplored NAD-RNAs and their hidden features in the *Drosophila* life cycle, from embryo to adult. Consistent with the finding in *Arabidopsis* and mouse,^11^^,^^14^ NAD capping in *Drosophila* tends to occur on genes with shorter length. Furthermore, we note that fly transcripts producing NAD-RNAs are likely to have shorter 5′ and 3′ UTR than those only capped by m^7^G. UTRs serve pivotal roles in gene expression control via *cis*-regulatory elements as well as RNA secondary structures.^20^ Reduction in the length of UTRs could not only affect the number of *cis*-regulatory motifs but also diminish the accessibility of *trans* effectors, e.g., RNA binding proteins, miRNAs targeting sites, and etc.^21^ The tendency of NAD-capping on those genes with shorter UTRs might provide a compensatory mechanism to fine-tune gene expression at the 5′ end. More interestingly, we discover that chromosomal clustering can couple co-expression with NAD capping on physically linked genes. Unlike prokaryotic operons, however, genes in eukaryotic chromosomes are transcribed independently but are, in many cases, organized in physical clusters, i.e., their chromosomal locations are adjacent to each other, and the clustered genes are co-expressed.^4^^,^^22^ NAD, an adenine-containing metabolite, can be added to the 5′-end of RNA during transcription initiation by RNA Pol II. We propose that RNA Pol II, as a common writer, drives the expression and capping of physical clustered genes, and this process may be influenced by the NAD level. However, it remains enigmatic how RNA Pol II can distinguish gene length and chromosomal structure at the step of transcription initiation.

We reveal features of gene expression and NAD capping during *Drosophila* metamorphosis transition. As fly develops from one stage of its life cycle to the next, NAD-RNA epitranscriptomes exhibit signature changes, with stage-specific NAD-RNAs and their distinguished biological pathways being involved. Moreover, we note that many ncRNA genes, especially at the pupa stage, possess NAD-capped forms, though the nature of NAD capping on those transcripts remains unknown. Moreover, comparative analysis identifies that the genes of chitin-based cuticle development display an inverse correlation between RNA expression and NAD modifications (i.e., high NAD capping at the pupa and adult stages correlate with low gene expression, whereas those genes are abundantly expressed at both embryo and larva when NAD capping is low). Since it has been reported that NAD cap can trigger RNA decay in cells,^23^^,^^24^^,^^25^ this observation prompts the hypothesis that increased NAD capping for cuticle-related genes might assist in shutting down gene expression and consequently promote the cuticular transformation from early to late developmental stages as flies eclose. As such, NAD modification may represent a novel mechanism underlying chitin-based cuticle development in *Drosophila*.

Taken together, we propose DO-seq as a simple, specific, and highly sensitive method, when combined with enONE algorithm, enabling quantitative profiling of NAD-RNAs and their dynamics from complex biological processes. With DO-seq, we are able to reveal hidden features of NAD-RNA epitranscriptome in *Drosophila* life cycle, laying critical foundation for further research to elucidate the molecular function and biological insight of NAD-capped RNAs.

### Limitations of the study

The current DO-seq platform proposed by this study mainly focuses on NAD-capped polyadylated RNAs; future study may broaden the scope into non-polyadylated RNAs by using the total RNA-seq strategy. Our current study describes hidden features of NAD-capped epitranscriptomes during *Drosophila* metamorphosis transition, which lacks mechanistic insights.

## STAR★Methods

### Key resources table

**Table:** 

REAGENT or RESOURCE	SOURCE	IDENTIFIER
**Chemicals, peptides, and recombinant proteins**		

yDcps	New England Biolabs	M0463S
Glycoblue	Thermo	AM9516
RRI	Takara	2313A
TRizol	Takara	9109
CIAP	Thermo	18009–027
NudC Pyrophosphatase	New England Biolabs	M0607S

**Critical commercial assays**		

Fast RNA-seq Lib Prep Kit V2	Abclonal	RK20306
MEGAscript T7 Transcription Kit	Thermo Fisher Scientific	AM1334
ABScript II cDNA First-Strand Synthesis Kit	ABclonal	RK20400
ABScript III RT Master Mix for qPCR	ABclonal	RK20428
T4 RNA Ligase 1 (ssRNA Ligase)	New England Biolabs	M0204S

**Deposited data**		

Raw and analyzed data	This paper	GSE228505

**Experimental models: Organisms/strains**		

*D. melanogaster*: 5905	Bloomington Drosophila Stock Center	RRID:BDSC_5905
Mouse: C57BL/6J	Charles River Laboratories	MGI:3028467

**Oligonucleotides**		

List of primers and other oligonucleotides, see Table S5	This paper	N/A

**Software and algorithms**		

enONE (v0.1.0)	Li et al., 2023 (https://doi.org/10.1101/2023.03.23.534034)	https://doi.org/10.5281/zenodo.8315820

### Resource availability

#### Lead contact

Additional information and requests for resources and reagents used in this study should be directed to and will be fulfilled by the lead contact, Nan Liu, (liunan@sioc.ac.cn).

#### Materials availability

This study did not generate new unique reagents.

#### Data and code availability

• All high-throughput RNA sequencing data as well as transcript quantifications have been deposited at the Gene Expression Omnibus (GEO) under accession number GSE228505.
• All original code has been deposited at Zenodo, https://doi.org/10.5281/zenodo.8328809. The datasets and the code are publicly available as of the date of publications.
• Other items: No other new unique reagent was generated. Any additional information required to reanalyze the data reported in this paper is available from the lead contact upon request.

### Experimental model and study participants details

Experiments were performed in C57BL/6 male mice aged 18-month and *D*. *melanogaster* (Bloomington stock 5905) with defined developmental phases. C57BL/6 mice were housed in an environmentally controlled room under a 12:12 h light/dark cycle at 23°C and were fed with commercial mouse chow food and water *ad libitum*. Flies were cultured in standard media at 25°C with 60% humidity in a light 12 h and dark 12 h cycle. All animal procedures have been reviewed and approved by the Institutional Animal Care and Use Committee at the Chinese Academy of Sciences and were in accordance with the Guide for the Care and Use of Laboratory Animals of the Chinese Academy of Sciences. All efforts were made to minimize the suffering of the animals.

### Method details

#### Mouse samples

To examine the efficacy of yDcpS-catalyzed clearance of m^7^G cap, livers were dissected from 18-month old male C57BL/6 mice. To assess the sensitivity of DO-seq, livers were dissected from 2-month old male C57BL/6 mice. Dissected livers were immediately frozen in liquid nitrogen.

#### *In vitro* transcription of pppA-RNA, NAD-RNA, and m^7^GpppA-RNA

To prepare m^7^GpppA-RNA of 38 nucleotides (nt), oligonucleotide without adenine was synthesized (Genewiz) and annealed to make double-stranded DNA template. (template sequence: 5′-GATCAC**TAATACGACTCACTATT**ACTGTGTCGTCGTCGTCTGCTGTCTCTCTCTCGCGGGC-3′; boldface letters denote the sequence of T7 class II promotor (ɸ2.5)) and (anti-sense: 5′-GCCCGCGAGAGAGAGACAGCAGACGACGACGACACAGTAATAGTGAGTCGTATTAGTGATC-3′). To prepare 45 nt NAD-RNA, oligonucleotide was synthesized (Genewiz) and annealed to make double-stranded DNA template. Primer extension was used to increase the length of the template to encode 45 nt RNA (template sequence: 5′-GATCAC**TAATACGACTCACTATT**ACTGTGTCGTCGTCGTCTGCTGTCTCTCTCTCGCGGGCCCTGTCG-3’; boldface letters denote the sequence of T7 class II promotor (ɸ2.5)) and (anti-sense: 5′- CGACAGGGCCCGCGAGAGAGAGACAGCAGACGACGACGACACAGTAATAGTGAGTCGTATTAGTGATC-3'). To prepare spike-in RNA of 106 nt, 88 nt oligonucleotide without adenine was synthesized (Genewiz) and annealed to make double-stranded DNA template. Primer extension was used to increase the length of the template to encode 106 nt RNA (template sequence: 5′-GATCAC**TAATACGACTCACTATT**ACTGTGTCGTCGTCGTCTGCTGTCTCTCTCTCGCGGGCGTGGGCGTGCGCGCTGCCGCCTGCGGCTCGCTGGCCCTGTCGTTCTTCTGCTCCTCCGGCTTGCTGCC-3'; boldface letters denote the sequence of T7 class II promotor (ɸ2.5)) and (anti-sense: 5′-GGCAGCAAGCCGGAGGAGCAGAAGAACGACAGGGCCAGCGAGCCGCAGGCGGCAGCGCGCACGCCCACGCCCGCGAGAGAGAGACAGCAGACGACGACGACACAGTAATAGTGAGTCGTATTAGTGATC-3'). To prepare spike-in NAD-RNA (500nt) and m^7^GpppA-RNA (500nt) with identical sequence, oligonucleotide without adenine was synthesized (Genewiz) and were subjected to polyadenylation for poly(A) tails elongation (template sequence: 5′-**TAATACGACTCACTATT**ATGGTGTGCTTGGGCGTGGTGCTGTTCTCCGGGGTGGTGCCCTTCCTGGTCGTGCTGGTCGGCGTCGTTTTCGGCCTCTTGTTCTGCGTGTCCGGCGTGGGCGTGGGCGTTGCCTCCTTCGGCTTGCTGTCCCTGTTGTTCTTCTGCTCCTCCGGCTTGCTGCCCGTGCCCTGGCCCTCCCTCGTGTCCTCCCTGTCCTTCGGCGTGCTGTGCTTCTGCCGCTTCCCCGTCCTCTTGTTGCTGCTCGTCTTCTTCTTGTCCGCCTTGCCCGTTGGCTTCGTCCTGGTGCGCTCCTTCTTCTTCTTGGTCGTCGGCTTCTTCTTGTCCCGCGCCGTGGTGTTGTTCGTGGGCGTCTCCCTGGTGTTCCGCTTCGTGCTGTTGGGCTTCGTCTTCTTGGTGGTCGGCTTCTTCCTGGGGCTCTTGCTGGTGTTCTTCTTCTTCTGCCTCTTCGTCTTTTTCTTGGCCGTCTTGCTGTTGTTCGGCTTCTTGGTGTTCTTCTT-3'; boldface letters denote the sequence of T7 class II promotor (ɸ2.5)) and (antisense: 5′-AAGAAGAACACCAAGAAGCCGAACAACAGCAAGACGGCCAAGAAAAAGACGAAGAGGCAGAAGAAGAAGAACACCAGCAAGAGCCCCAGGAAGAAGCCGACCACCAAGAAGACGAAGCCCAACAGCACGAAGCGGAACACCAGGGAGACGCCCACGAACAACACCACGGCGCGGGACAAGAAGAAGCCGACGACCAAGAAGAAGAAGGAGCGCACCAGGACGAAGCCAACGGGCAAGGCGGACAAGAAGAAGACGAGCAGCAACAAGAGGACGGGGAAGCGGCAGAAGCACAGCACGCCGAAGGACAGGGAGGACACGAGGGAGGGCCAGGGCACGGGCAGCAAGCCGGAGGAGCAGAAGAACAACAGGGACAGCAAGCCGAAGGAGGCAACGCCCACGCCCACGCCGGACACGCAGAACAAGAGGCCGAAAACGACGCCGACCAGCACGACCAGGAAGGGCACCACCCCGGAGAACAGCACCACGCCCAAGCACACCATAATAGTGAGTCGTATTA-3′).

For annealing oligonucleotides, 10 μM template oligo DNA were combined in 50 μL of 1× Cut Smart buffer (New England Biolabs, catalog: B7204S) and annealed by heating to 95°C for 5 min. The annealing reaction was by cooling down to 4 °C at a ramp rate of 0.1°C/s in a thermocycler (Eppendorf, Germany).

For *in vitro* transcription,10 μM of double-stranded DNA (dsDNA) template in 100 μL 1×transcription buffer (Thermo Fisher Scientific, catalog: AM1334), along with 1 mM of each of GTP, CTP and UTP, with 1mM ATP (for pppA-RNA), 4 mM NAD (for NAD-RNA) or 4 mM m^7^GpppA (New England Biolabs, catalog: S1406S) (for m^7^G-RNA), 10 μL of T7 RNA polymerase (Thermo Fisher Scientific, catalog: AM1334), 5% DMSO, 5 mM DTT and 40 U RNase Inhibitor (Takara Bio, catalog: 2313B) were added and the transcription mixture was incubated at 37°C for 4 h. The reaction was incubated with 10 U DNase I (Thermo Fisher Scientific, catalog: AM1334) at 37°C for 30 min to remove the DNA template. RNA was then purified using RNAiso Plus (Takara Bio, catalog: 9108) and precipitated by isopropanol with 0.3 M sodium acetate (Thermo Fisher Scientific, catalog: AM9740) at −80°C overnight. The RNA pellet was washed twice with 75% ethanol, air-dried, redissolved in DEPC-treated H2O, and stored at −80°C.

#### yDcpS treatment of NAD-RNA (45 nt) and m^7^GpppA-RNA (38 nt)

300 ng of 45 nt NAD-RNA and 300 ng of 38 nt m^7^GpppA-RNA were mixed together and incubated with 4 U yDcpS (New England Biolabs, catalog: M0463S) in 50 μL of yDcpS reaction buffer (10 mM Bis-Tris-HCl, 1 mM EDTA) at 37°C for 30 min. The product was purified with RNAiso Plus (Takara Bio, catalog: 9108) and analyzed by an 8% polyacrylamide TBE-Urea gel. Gel was stained by SYBR Gold (Invitrogen, catalog: S11494) and fluorescence was detected by Fusion Solo S imaging system (Vilber).

#### HEEB reaction with 106 nt spike-in RNAs

600 ng of NAD-RNA (106 nt), pppA-RNA (106 nt) and m^7^GpppA-RNA (106 nt) were separately incubated with 200 mM HEEB (1 M stock in DMSO) and ADPRC (25 μg/mL) at 37°C for 1 h. The products were separated into two aliquots. One aliquot was continued to incubate at 25°C for 30 min with 6 μL streptavidin magnetic beads (MedChemExpress, catalog: HY-K0208), which were pre-blocked with 100 μg/mL acetylated bovine serum albumin in 100 μL of 1×immobilization buffer (1 M NaCl, 10 mM Na-Hepes (pH 7.0), and 5 mM ethylenediaminetetraacetic acid). 20 μL RNA loading buffer was used to boil the RNAs off the beads. Two aliquots were denatured at 94°C for 2 min and further analyzed by 8% polyacrylamide TBE-Urea gel. Gel was stained by SYBR Gold (Invitrogen, catalog: S11494) and imaged by Fusion Solo S imaging system (Vilber).

#### DO-seq: yDcpS-catalyzed pre-treatment

Total RNAs from mouse liver tissues and *Drosophila* samples were isolated in accordance with the manufacturer’s instruction (Takara Bio, catalog: 9108). The mixture of total RNAs and spike-in RNAs was incubated with 4 U yDcpS (New England Biolabs, catalog: M0463S) and 20 U RNase Inhibitor (Takara Bio, catalog: 2313B) in 50 μL of yDcpS reaction buffer (10 mM Bis-Tris-HCl, 1 mM EDTA) at 37°C for 50 min 350 μL RNAiso Plus (Takara Bio, catalog: 9108) was added to stop the reaction. After thoroughly mixing with 80 μL chloroform, samples were centrifuged for 15 min at 12,000 × g at 4°C. The upper aqueous phase containing RNAs was kept. 1 volume of isopropanol and 0.1 volume of 3 M pH 5.5 sodium acetate (Thermo Fisher Scientific, catalog: AM9740) were added to precipitate RNAs. RNA pellets were washed twice with 75% ethanol, air-dried and re-dissolved in 50 μL of DEPC-treated H_2_O.

#### DO-seq: ADPRC-catalyzed HEEB reaction

After yDcpS-catalyzed pretreatment, samples were incubated with 200 mM HEEB (1 M stock in DMSO), 25 μg/mL ADPRC and 40 U RNase Inhibitor (Takara Bio, catalog: 2313B) in 100 μL of ADPRC reaction buffer (50 mM Na-HEPES pH 7.0, 5 mM MgCl_2_) at 37°C for 1 h. 100 μL of DEPC-treated H2O was then added and acid phenol/ether extraction was performed to stop the reaction.^8^ Similarly, 1 volume of isopropanol and 0.1 volume of 3 M pH 5.5 sodium acetate (Thermo Fisher Scientific, catalog: AM9740) were added to precipitate RNAs from the aqueous phase. RNA pellets were washed twice with 75% ethanol, air-dried and re-dissolved in 54 μL of DEPC-treated H_2_O. 4 μL of biotinylated RNAs were kept as input and the left RNAs proceeded to an enrichment procedure.

#### DO-seq: Enrichment of biotinylated NAD-RNA

After HEEB reaction, RNAs were incubated at 25°C for 30min with 6 μL streptavidin magnetic beads (MedChemExpress, catalog: HY-K0208), which were pre-blocked with 100 μg/mL acetylated bovine serum albumin in 100 μL of immobilization buffer containing NaCl (1 M), Na-Hepes (10 mM, pH 7.0), and ethylenediaminetetraacetic acid (5 mM). Beads were then washed five times with streptavidin wash buffer containing urea (8 mM) and Tris-HCl (50 mM, pH 7.4), and once with DEPC-treated H2O. Biotinylated RNAs were finally eluted by incubating the beads with 20 μL of 10 mM biotin solution at 94°C for 8 min. Beads were washed once with 30 μL DEPC-treated H2O which is then combined with 20 μL the biotin elution. 50 μL biotin-eluted RNAs were subjected to library construction.

#### DO-seq: polyA-selected RNA sequencing

Input (see above) and biotin-eluted RNAs were used for NGS library construction, in accordance with the manufacturer’s instructions (Fast RNA-seq Lib Prep Kit V2, Abclonal, catalog: RK20306). Library quality was assessed by Bioanalyzer 2100 (Agilent, United States), and quantification was performed by qRT-PCR with a reference to a standard library. Libraries were pooled together in equimolar amounts to a final 2 nM concentration and denatured with 0.1 M NaOH (Sigma, catalog: 72068). Libraries were sequenced on the Illumina NovaSeq 6000 system (paired-end; 150 bp).

#### qRT-PCR for 106 nt spike-in RNA

Total RNAs (50 μg) were mixed with 50 ng spike-in RNAs (106 nt) for each sample. First, samples were incubated with or without 4 U yDcpS (New England Biolabs, catalog: M0463S) in 50 μL of yDcpS reaction buffer (10 mM Bis-Tris-HCl, 1 mM EDTA) at 37°C for 30 min, followed by RNA purification by RNAiso Plus (Takara Bio, catalog: 9108). Second, samples were incubated with HEEB (200 mM for NAD-RNA and pppA-RNA, 400 mM for m^7^GpppA-RNA) with or without ADPRC (25 μg/mL) in 100 μL of ADPRC reaction buffer (50 mM Na-HEPES pH 7.0, 5mM MgCl2) at 37°C for 1 h, followed by acid phenol/ether extraction. Next, samples were incubated at 25°C for 30min with 6 μL pre-blocked streptavidin magnetic beads (MedChemExpress, catalog: HY-K0208) and then washed five times with streptavidin wash buffer (8 mM urea, 50 mM Tris-HCl pH 7.4), and once with DEPC-treated H2O. Biotinylated RNAs were finally eluted by incubating the beads with 20 μL of 10 mM biotin solution at 94°C for 8 min. Biotin-eluted RNAs from three biological replicates were used for qRT-PCR. Reverse transcription was performed with gene specific primers using SuperScript III SuperMix (Vazyme, catalog: R323-01). qPCR was performed using SYBR Green master mix (Vazyme, catalog: Q111-02) to detect NAD-RNA, ppp-RNA or m^7^GpppA-RNA from three biological replicates using specific primers. Significance was assessed by Student’s *t* test. NAD-RNA (106 nt)/m^7^GpppA-RNA (106 nt): Forward primer: 5′-ACTGTGTCGTCGTCGTCTGC-3′, Reverse primer: 5′-GGCAGCAAGCCGGAGGAGCA-3'; pppA-RNA (106 nt): Forward primer: 5′-GGATACCGGGAAAACGCTGG-3′, Reverse primer: 5′-GTTGGTCGCTTCCGGATTG-3').

#### DO-seq with polyadenylated 500 nt spike-in RNA

Total RNAs (100 μg) were mixed with 1 ng of 3′-end polyadenylated spike-in RNA (500 nt) that had 0% NAD-RNA/100% m^7^GpppA-RNA, 1% NAD-RNA/99% m^7^GpppA-RNA or 5% NAD-RNA/95% m^7^GpppA-RNA, respectively. The mixtures were incubated with 200 mM HEEB (1 M stock in DMSO) with ADPRC (25 μg/mL) in 100 μL of ADPRC reaction buffer (50 mM Na-HEPES pH 7.0, 5 mM MgCl2) at 37°C for 1 h, following yDcpS-catalyzed pre-treatment. Input (see above) and biotin-eluted RNAs from three biological replicates of livers from 2-month old C57BL/6 mice were subjected to polyA-selected RNA sequencing as mentioned above.

#### Collection of *drosophila* samples

To get fly samples with defined developmental phases, 3-7days old male and virgin flies were crossed overnight for adaptation before collection. For late-stage embryos (AEL 14-16h) collection, adults were first allowed to do a 2h prelay with apple juice plates supplemented with yeast paste to remove old embryos.^26^ Then flies were set for 2 h of egg laying on fresh plates. Plates were collected and aged for 14h. Subsequently, embryos were harvested from the plates, dechorionated in 50% bleach solution for 2 min, and washed once in 1X Phosphate Buffered Saline supplemented with 0.1% Tween 20 (PBT) and twice in deionized water.^27^ For well-staged larva and pupa collection, mated adults were transferred to new, fresh vials every 4 h. Vials were collected and aged for 96h and 7days separately. To obtain larva from vials, 1.5 M NaCl was added to let the larva float to the top. Pupa were carefully picked up by a forcep. Both larva and pupa were washed by 1X PBS twice. For 3days old adults collection, new-born flies were collected after ecolsion and separated as males and virgin females into new vials, followed by 72 h of aging. Mixed 3days sample consisted of equivalent amounts of male and virgin flies. All the samples were frozen in liquid nitrogen and stored at −80°C.

#### Identification of physical clusters of genes on the chromosomes

The method was modified from the previous research.^28^ We defined a physical cluster as a group of ***N*** (***N*** ≥ 3) genes producing NAD^+^ capped transcripts where the distance between two adjacent genes was not over ***L*** kilobases. The distance was calculated as the difference between the stop position of the most 5′ gene and the start position of the most 3′ gene. A cluster was formed or expanded disregarding the orientation of the genes or the existence of genes not producing NAD capped transcripts. To interrogate whether the number of physical clusters identified from ***n*** genes with the given ***L*** is more than what we expect to see randomly, the number of clusters found in the true dataset, **C**_**true**_ was compared with the null distribution **C**_**random**_. To get the null distribution, **C**_**random**_, we repeated the process of cluster identification on ***n*** randomly chosen genes with the same chromosomal distribution and ***L*** setting for 10000 times. The p-value of the true datasets was the number of **C**_**random**_ ≥ **C**_**true**_ divided by 10000. If **C**_**true**_ > **C**_**random**_ for all times, the p-value was less than 0.0001.

#### Validation of NAD-capped RNA by a modified CapZyme-Seq method

Total RNAs were extracted from four biological replicates from 3days *Drosophila* samples as described above. For each replicate, total RNAs (20 μg) were mixed with polyadenylated spike-in RNAs (1 ng NAD-RNA 500nt, 1 ng m^7^Gppp-RNA 500nt, and 1 ng of ppp-RNA 500nt). RNAs were incubated with 20 U CIAP (Thermo Fisher Scientific, catalog: 18009019) in the presence of 40 U RNaseOUT at 37°C for 1 h to remove 5′-terminal phosphate from non-capped RNAs. After purification with RNAiso Plus (Takara Bio, catalog: 9108), CIAP-treated RNAs were incubated with 250 nM NudC (New England Biolabs, catalog: M0607S) in 40 μL of NudC reaction buffer (100 mM NaCl, 50 mM Tris–HCl pH 7.9, 10 mM MgCl2, 100 μg/mL Recombinant Albumin, 40 U RNaseOUT) at 37°C for 30 min. This step aims to hydrolyze the cap from NAD-RNA resulting in ligatible monophosphate at the 5′ end. Products were purified with RNAiso Plus (Takara Bio, catalog: 9108) and further ligated with 100 μM 5′ adaptor oligo listed in Table S5, in the presence of 15 U T4 RNA ligase 1 (New England Biolabs, catalog: M0202), 15% of PEG8000, 1 mM ATP and 40 U RNaseOUT in 40 μL of 1× T4 RNA ligase buffer at 16°C for 16 h. RNAs were purified and re-dissolved in 20 μL of DEPC-treated H_2_O. Reverse transcription was performed with designed primer listed in Table S5, by ABScript II cDNA First-Strand Synthesis Kit (ABclonal, catalog: RK20400). 10 μL of products was diluted to 200 μL and served as input. Meanwhile, 5 μL of reversely transcribed products were further amplified by PCR using adaptor-specific primers listed in Table S5. The PCR program operated with an initial denaturation step of 3 min at 95°C, amplification for 14 cycles (denaturation for 15 s at 95°C, annealing for 15 s at 60°C and extension for 6 min at 72°C), and a final extension for 5 min at 72°C. While ppp-RNA (500 nt) was served as the baseline, NAD-RNA (500 nt) was used as a positive control, and m^7^G-RNA (500 nt) was used as a negative control. Primers were listed in Table S5. To calculate the enrichment from qRT-PCR data, the Ct value of the target gene was first normalized to the Ct of the ppp-RNA (baseline). Next, the normalized PCR+ fraction value (ΔCt of the target gene normalized to the ppp-RNA) was normalized to the background (ΔCt calculation for the gene in the input), to yield the ΔΔCt value. The linear conversion of this ΔΔCt resulted in the fold enrichment. Significance was assessed by Student’s *t* test.

### Quantification and statistical analysis

DO-seq sequencing reads were trimmed for adapter sequence by Trim Galore (v0.6.6)^29^ and remapped to *Drosophila melanogaster* genome r6.36 and *Mus musculus* genome GRCm39 simultaneously using STAR (2.7.6a)^30^ aligner. The read counts for each gene were calculated by featureCounts (v2.0.1)^31^ with parameters “-p -B -C”. mRNA-seq sequencing reads were processed through the same procedures except that trimmed reads were only mapped to drosophila reference genome r6.36. Sequencing saturation was assessed by statistical analysis of gene number with over 10 read counts after random subsampling of the library. Counts normalization and NAD-RNAs identification were done by R package enONE.^12^ DESeq_RUVs_k2 was selected as the final normalization method. Gene annotation information including gene biotype, chromosomal location, start/stop coordinate and 5’/3′ UTR were fetched via R package biomaRT (v2.48.3).^32^ 5′ and 3′ UTR length for each gene was defined as the longest one. GO analysis was performed using clusterProfiler package (v4.5.2.001).^33^ Pie chart, barplot, boxplot and scatterplot were created via R package ggpubr (v0.4.0)^34^ and ggplot2 (v3.3.6).^35^ Venn diagram and upset plot were generated by R package eulerr (v6.1.1)^36^ and UpSetR (v1.4.0)^37^ respectively. *Z* score transformation and Pearson correlation analysis were calculated by using the bulit-in functions in R. Double y axis figures were produced by GraphPad Prism (v8.3.0). Heatmaps were plotted by R package pheatmap.^38^ Examples of physical gene clusters were drawn by R package gggenes (v0.4.1)^39^ and ggplot2 (v3.3.6).^35^ Statistical analyses were performed using either a two-tailed t-test or one-way ANOVA. Specific statistical tests used, significance, number of animals used and other details were indicated in individual figure legends.

## Supplemental information

Document S1. Figures S1–S 5

Table S1. Gene lists demonstrate noise canceling effect of yDcpS to reduce potential false signals from m7G-capped RNAs, related to Figure 1E (A) The list of genes enriched by yDcpS+ group. (Fold Change ≥2, the adjusted p-value <0.05) (B) The list of genes enriched by yDcpS- group. (Fold Change ≥2, the adjusted p-value <0.05).

Table S2. Gene lists of newly-identified NAD-capped RNAs by DO-seq from embryo, larva, pupa and adult fly (3days), related to Figure 3B (A) NAD-RNA identified by DO-seq from *Drosophila* at embryo, (B) larva, (C) pupa, (D) adult (3days) stage (Fold Change ≥2, the adjusted p-value <0.05). (E) NAD-RNA from distinct developmental stages of *Drosophila* by improving the screening criteria, such as Fold Change ≥4, (F) Fold Change ≥6, (G) Fold Change ≥8.

Table S3. Clusters identified from four datasets when L = 3 kb, related to Figure 4 and STAR Methods (A) Details of clusters identified from *Drosophila* at embryo, (B) larva, (C) pupa, (D) adult (3days) stage respectively.

Table S4. Gene lists of transcription factor and cofactor capped by NAD, related to Figures 5A and S4 (A) List of genes corresponding to transcription factor with NAD-cap (172 genes). (B) List of genes corresponding to transcription cofactor with NAD-cap (102 genes).

Table S5. Sequences of primers and other nucleotides used for qRT-PCR analysis of selected genes for data validation, related to STAR Methods, Figures S3 and S1D (A) qPCR primers for data validation. (B) Oligonucleotides used in modified CapZyme-seq method for validation of NAD-RNA.
