# Supplementary material for: Hidden features of NAD-RNA epitranscriptome in Drosophila life cycle
Source: iScience. 2023 Dec 2;27(1):108618. doi: 10.1016/j.isci.2023.108618 (PMC10775904; doi:10.1016/j.isci.2023.108618)
Supplement: Document S1. Figures S1–S — 5 [file mmc1.pdf]

iScience, Volume *27*

Supplemental information

Hidden features of NAD-RNA

epitranscriptome in *Drosophila* life cycle

Shuwen Ge, Xueting Wang, Yingqin Wang, Minghui Dong, Dean Li, Kongyan Niu, Tongyao Wang, Rui Liu, Chao Zhao, Nan Liu, and Ming Zhong

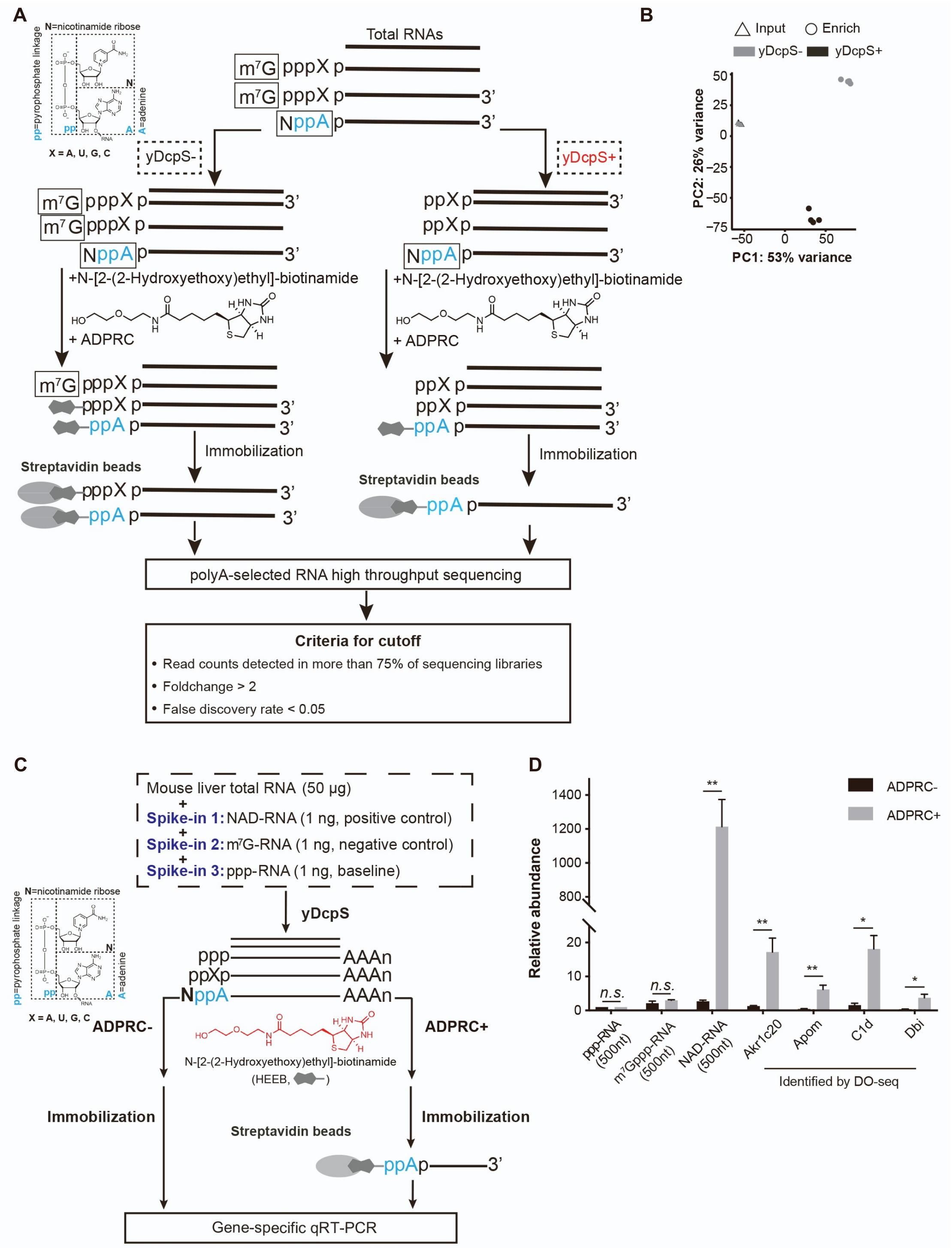

Figure S1. Assessment of DO-seq, related to Figure 1

(A) The overview to test noise-canceling effect of yDcpS. Total RNAs, either mock-treated or yDcpS-treated, were subjected to HEEB reaction, followed by enrichment via streptavidin beads. (B) Principal component analysis indicating the high quality and reproducibility of the sequencing data. (C) The workflow for assessing the specificity of capture procedures using qRT-PCR. (D) Assessment of gene-specific NAD-capping by qRT-PCR with or without ADPRC. Specific genes such as Akr1c20, Apom, C1d and Dbi, identified as NAD-RNAs, were examined. (Two-tailed Student's t test: $^{**}P< 0.01$, $^{*}P< 0.05$; *n.s.*, not significant).

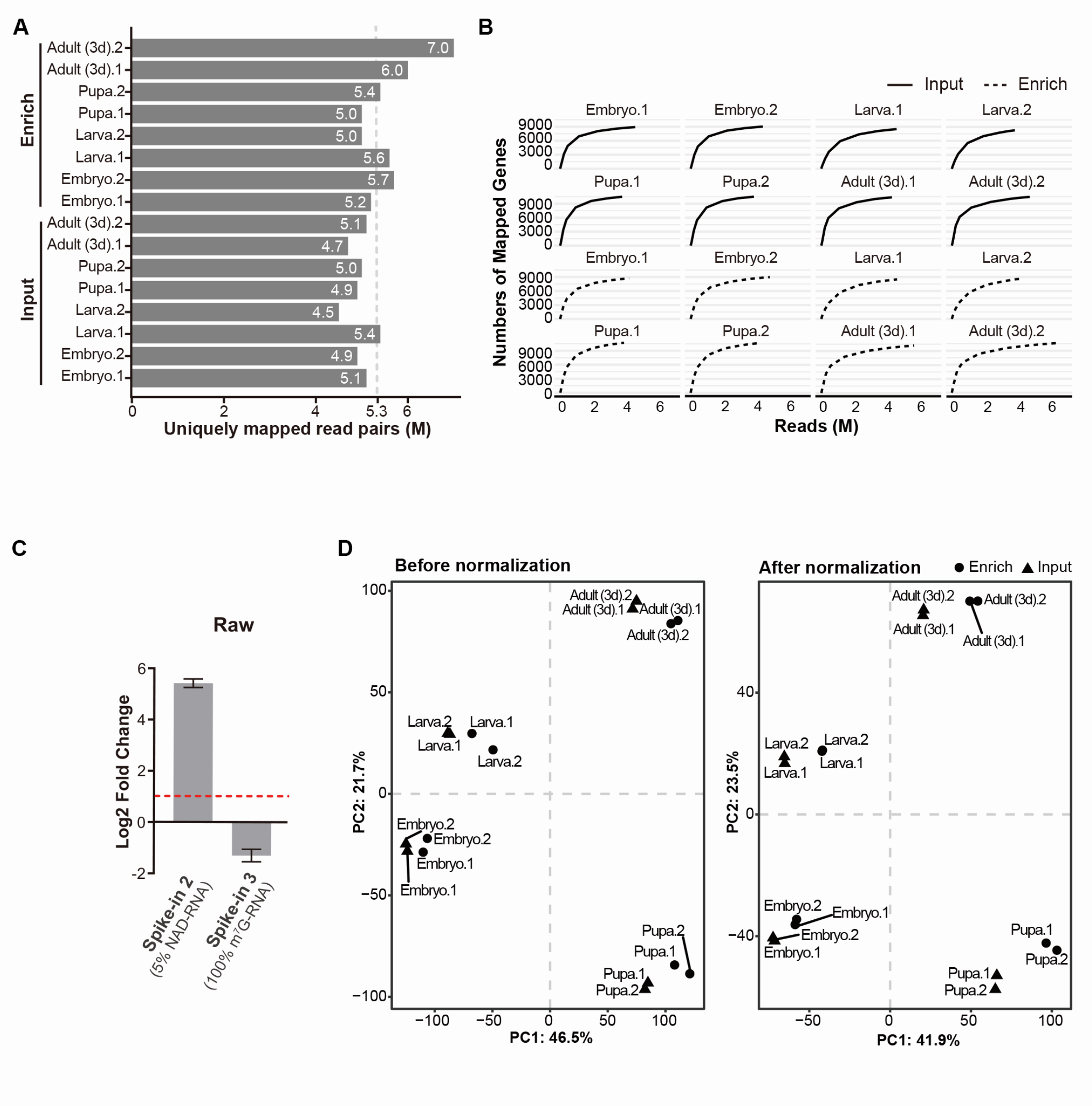

Figure S2. Quality control and normalization of sequencing data, related to Figure 3

(A) Analysis of sequences alignment from fly samples. (B) Analysis of sequencing saturation. RNA samples as in (A). (C) Enrichment level of spike-ins. Spike-in 2, which contained 5% NAD-capped forms, was significantly enriched, whereas no enrichment was found for spike-in 3 made up with 100% m^7G-RNA. The cutoff (log2 Fold Change = 1) was indicated as red dashed line. Data were shown in mean ± s.e.m. (D) PCA illustrating high reproducibility of experiments upon enONE normalization.

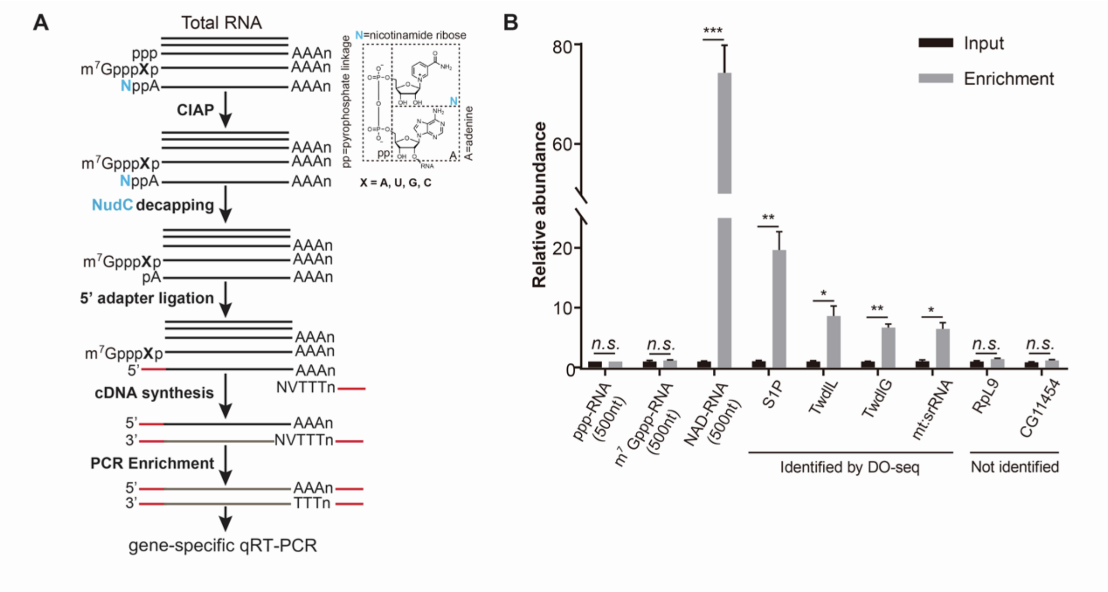

Figure S3. Validation of NAD-RNAs by modified CapZyme-Seq, related to Table S5

(A) The workflow of the validation strategy. (B) Assessment of gene-specific NAD-capping by qRT-PCR. S1P, TwdlL, TwdlG, and mt:srRNA identified by DO-seq as well as RpL9 and CG11454 not identified by DO-seq were examined. Data were shown in mean ± s.e.m. (Two-tailed Student's *t* test: ***P < 0.001, **P< 0.01, *P< 0.05; *n.s.*, not significant).

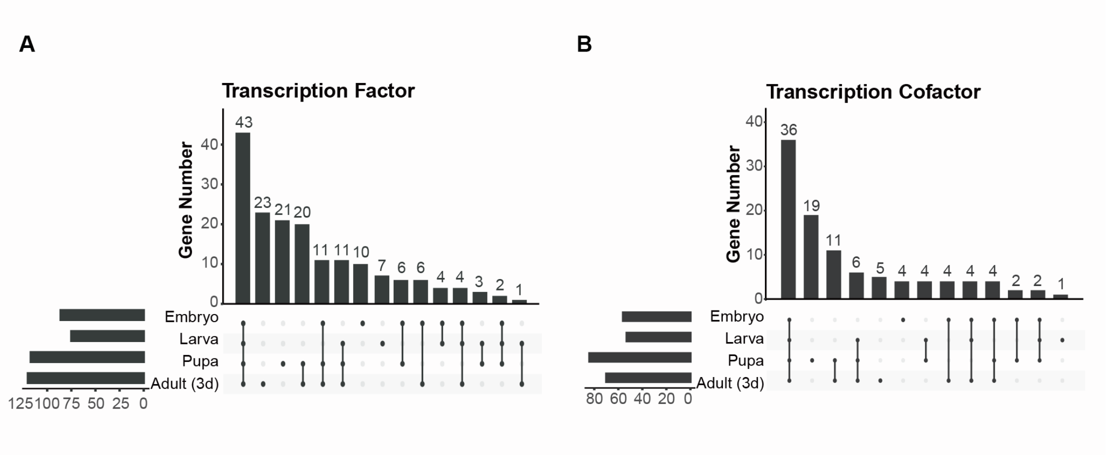

Figure S4. Upset plot depicting the number of unique and shared transcription factors (left panel) and cofactors (right panel) capped by NAD, related to Table S4

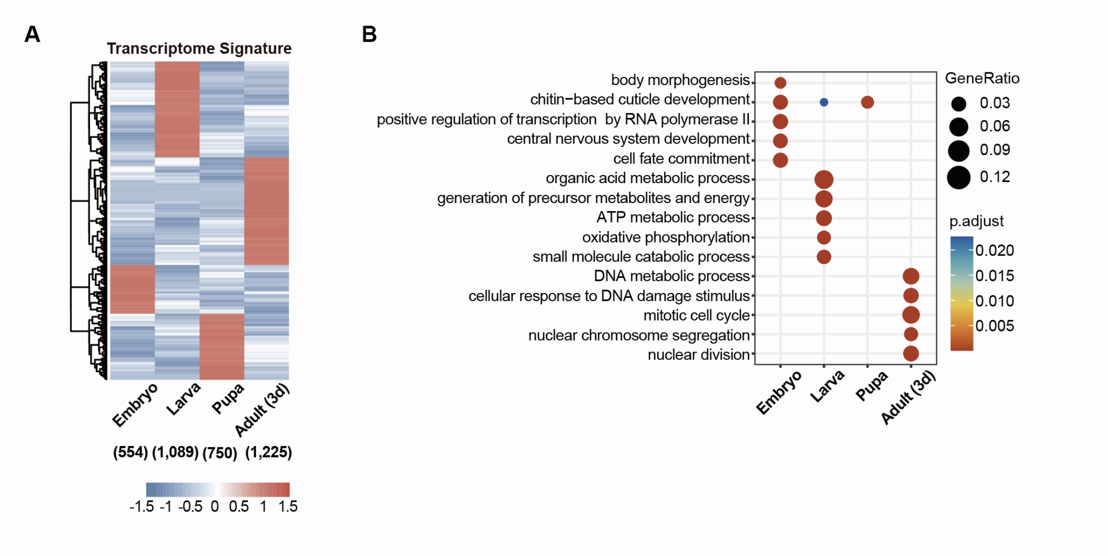

Figure S5. Transcriptome signatures for each stage, related to Figure 5

(A) Heatmap showing transcriptome signatures identified for each stage by combining ANOVA (FDR < 0.01) and z-score transformation (z-score > 1.3). (B) Pathway analysis noted that chitin-based cuticle development was enriched as transcriptome signature from embryo to pupa except for adult.
